# Implementation of multidimensional knowledge translation strategies to improve procedural pain in hospitalized children

**DOI:** 10.1186/s13012-014-0120-1

**Published:** 2014-11-25

**Authors:** Bonnie J Stevens, Janet Yamada, Sara Promislow, Jennifer Stinson, Denise Harrison, J Charles Victor

**Affiliations:** The Hospital for Sick Children and University of Toronto, 686 Bay Street, Room 06.9712, Toronto, M5G 1X8 Ontario Canada; Children’s Hospital of Eastern Ontario and University of Ottawa, 401 Smyth Road, Room R214, Ottawa, K1H 8 L1 Ontario Canada; Institute of Health Policy, Management, and Evaluation and University of Toronto, G1 06, 2075 Bayview Avenue, Toronto, M4N 3M5 Ontario, Canada; Members of the CIHR Team in Children’s Pain, Canada

**Keywords:** Pediatric procedural pain, Knowledge translation strategies, Tailored interventions, Quality improvement

## Abstract

**Background:**

Despite extensive research, institutional policies, and practice guidelines, procedural pain remains undertreated in hospitalized children. Knowledge translation (KT) strategies have been employed to bridge the research to practice gap with varying success. The most effective single or combination of KT strategies has not been found. A multifaceted KT intervention, Evidence-based Practice for Improving Quality (EPIQ), that included tailored KT strategies was effective in improving pain practices and clinical outcomes at the unit level in a prospective comparative cohort study in 32 hospital units (16 EPIQ intervention and 16 Standard Care), in eight pediatric hospitals in Canada.

In a study of the 16 EPIQ units (two at each hospital) only, the objectives were to: determine the effectiveness of evidence-based KT strategies implemented to achieve unit aims; describe the KT strategies implemented and their influence on pain assessment and management across unit types; and identify facilitators and barriers to their implementation.

**Methods:**

Data were collected from each EPIQ intervention unit on targeted pain practices and KT strategies implemented, through chart review and a process evaluation checklist, following four intervention cycles over a 15-month period.

**Results:**

Following the completion of the four cycle intervention, 78% of 23 targeted pain practice aims across units were achieved within 80% of the stated aims. A statistically significant improvement was found in the proportion of children receiving pain assessment and management, regardless of pre-determined aims (p < 0.001). The median number of KT strategies implemented was 35 and included reminders, educational outreach and materials, and audit and feedback. Units successful in achieving their aims implemented more KT strategies than units that did not. No specific type of single or combination of KT strategies was more effective in improving pain assessment and management outcomes. Tailoring KT strategies to unit context, support from unit leadership, staff engagement, and dedicated time and resources were identified as facilitating effective implementation of the strategies.

**Conclusions:**

Further research is required to better understand implementation outcomes, such as feasibility and fidelity, how context influences the effectiveness of multifaceted KT strategies, and the sustainability of improved pain practices and outcomes over time.

**Electronic supplementary material:**

The online version of this article (doi:10.1186/s13012-014-0120-1) contains supplementary material, which is available to authorized users.

## Background

Pain assessment and management practices for hospitalized children remain sub-optimal, despite significant research, best practice guidelines, and institutional policies. Knowledge translation (KT) strategies can be effective in promoting health care professionals’ use of clinical research evidence to enhance clinical practice and improve clinical outcomes [1]; however, translating evidence into practice is a complex process, involving behavioural change among health care professionals [2].

The implementation of single KT strategies (*e.g.*, reminders, educational materials, educational outreach, and audit and feedback) in the dissemination of practice guidelines has resulted in small to moderate improvements in patient care [3]. Based on the results of 33 systematic reviews, Prior *et al.*[4] reported that the most effective KT strategies included the use of multifaceted interventions, such as interactive education sessions and clinical reminder systems. However, some researchers have posited that multifaceted interventions may be no more effective than single strategies [1],[5]. To date, researchers have been unable to identify the most effective KT strategies or strategy combinations, as varying contexts require different strategies. Some studies highlight the importance of contextualizing strategies to meet this need [6].

The Evidence-Based Practice for Improving Quality (EPIQ) is a multifaceted intervention that can be tailored to achieve clinical aims of hospital units [7],[8]. EPIQ incorporates evidence-based KT strategies and continuous quality improvement methods to improve clinical practices and outcomes in hospitalized children. EPIQ comprises two phases: *Preparation Phase,* including the identification of pain practice change champions (health care professionals) at the unit level, who develop targeted practice change aims based on local baseline data and research evidence; *Implementation and Change Phase*, including planning, developing, implementing, and evaluating KT strategies to improve the targeted unit pain practices and monitoring practice change progress over time. EPIQ was first effectively implemented in neonatal intensive care units to reduce nosocomial infection rates and bronchopulmonary dysplasia [7]. EPIQ was adapted to reach a broader pediatric patient population and unit type and was further tailored; resulting in significantly improved acute pain assessment and management practices and clinical outcomes of hospitalized children across Canada [8].

**Figure 1:**
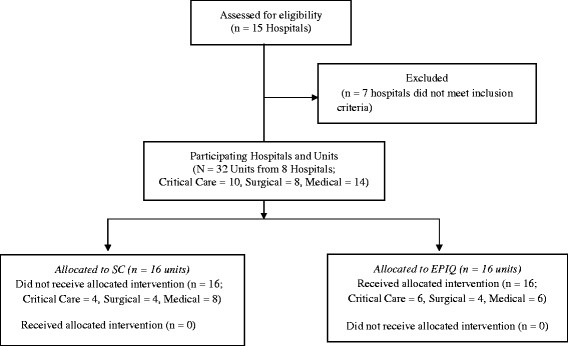
**Allocation diagram.** SC, Standard Care; EPIQ, Evidence-based Practice for Improving Quality.

The CIHR Team in Children’s Pain successfully implemented the EPIQ intervention in a prospective cohort comparative design study with repeated measures. Thirty-two inpatient hospital units, consisting of medical, surgical, and critical care units from eight Canadian tertiary, pediatric urban-based health centers, participated in the study over a 15-month intervention period. Details regarding the eligibility of participating units have been previously described [8]. Sixteen units (six medical, four surgical, six critical care) were allocated to receive the EPIQ intervention while the remaining 16 units (eight medical, four surgical, four critical care) received standard care. At each hospital, to achieve overall balanced allocation, two units were allocated to EPIQ and two to standard care. Allocation was based on units’ baseline pain assessment and management practices [9],[10], allowing for equal inclusion of both high- and low-performing units in the intervention and standard care groups (Figure 1).

Stevens *et al.*[8] reported that the proportion of patients whose pain was assessed, using validated assessment tools, significantly increased in the units that implemented EPIQ compared to standard care units (p < 0.001). The proportion of analgesics administered for procedures increased significantly more in the intervention group (p = 0.03). The use of physical pain management strategies also significantly increased over time in the intervention group (p = 0.02). These improvements in pain assessment and management practices were also associated with improved clinical pain outcomes. After controlling for patient and unit level factors, the odds of having severe pain were 51% less for those in the EPIQ units versus standard care units.

In the present study, a detailed examination was undertaken of the 16 EPIQ units only to:

1. determine the effectiveness of the evidence-based KT strategies implemented in relation to unit aims;
2. describe the KT strategies implemented and their influence on pain assessment and management practices across unit types; and
3. identify facilitators and barriers to the implementation of the KT strategies.

The overall goal was to improve pain assessment and management in hospitalized children over time.

## Methods

### Study design

This study focused on the 16 hospital units allocated to the EPIQ intervention. Demographic characteristics of the participating units are summarized in Table 1. Both qualitative and quantitative measures were employed. Data on the targeted pain assessment and management practices specified in the unit pain practice change aims were collected at baseline through chart review and following the completion of each of four, three-month intervention cycles during the EPIQ intervention. Data on the single and combinations of KT strategies used by each unit to achieve their aims were collected using a validated, process evaluation checklist [11] at the end of each of the four intervention cycles.

**Table 1:** **Evidence-based Practice for Improving Quality (EPIQ) intervention unit characteristics**

Type of unit	n (%)
- Surgical	4 (25.0)
- Medical	6 (37.5)
- NICU	2 (12.5)
- PICU	4 (25.0)
	**Mean (Standard Deviation)**
Age on unit, years	5.5 (3.4)
Total patient days	457.4 (296.7)
Patient stay, days	7.2 (4.3)
Occupied beds	17.8 (9.0)

### EPIQ intervention

The EPIQ intervention consisted of four, three-month intervention cycles with one month between each cycle. The intervention was informed by a Plan-Do-Study-Act framework [12],[13]. All EPIQ units followed the same process and timing of iterative cycles, where targeted pain practice aims were determined and planned (Plan step), developed and implemented (Do step), evaluated and monitored (Study step), and enhanced, revised or abandoned (Act step). Use of KT strategies for successive cycles was based on results of the end of cycle audits on the progress of practice change and feedback on the usefulness of KT strategies implemented. Within the intervention cycles, units chose to implement smaller audit and feedback strategies (*e.g.*, auditing of 5 – 10 medical records) among other KT strategies (*e.g.*, reminders, educational outreach) to achieve their practice change aims. A detailed explanation of the two phases of EPIQ follows.

In the Preparation Phase of EPIQ, a small group of multidisciplinary clinical staff members (*e.g.*, physicians, nurses, quality improvement personnel) were identified on each intervention unit to lead and champion EPIQ. The identification of champions and their motivation to participate in the intervention varied from unit to unit; for example, some were recommended by the unit manager, while others volunteered when learning about the intervention. These individuals formed the Research Practice Council. Effort was made to attain multidisciplinary representation. On average, each Research Practice Council included four to six members, with a total of 93 members across the 16 EPIQ units in the eight sites by the end of the study. Over one-half (56%) of the Research Practice Council members were nurses (*e.g.*, bedside nurses, nurse practitioners, nurse educators, clinical leaders, and managers); 19% were physicians (*e.g.*, staff and fellows); and 25% were from other professions (*e.g.*, rehabilitation and respiratory therapists, child life specialists, and pharmacists, among others).

The Research Practice Councils were locally trained by two research team members on the EPIQ intervention, including KT and quality improvement methods. The research team provided each of the Research Practice Councils with information on their unit’s baseline pain assessment and management practices. Informed by unit baseline data, the unit’s clinical priorities, and relevant evidence summaries provided by the core site, each Council identified and defined unique aims for improving pain practices on their unit. The aims were not predetermined by the research team or chosen from a list of potential aims. They were unique and tailored to their pain practice needs and preferences. For example, if a unit’s baseline data revealed that no validated pain assessment tools were employed, the unit would often choose implementing pain assessment as the focus of their practice change.

Based on relevant evidence summaries, the Council chose age appropriate, validated pain measures or pain management strategies to implement on their unit. Furthermore, each unit decided on the degree of improvement they sought to achieve in their targeted practices. Aims varied from a 25% increase in the use of a targeted practice to 100%. Examples of pain practice change aims included: increasing the use of pain management strategies for painful procedures (*e.g.*, local anesthetic agents, sucrose, and physical strategies, such as: non-nutritive sucking, kangaroo care, and facilitated tucking); increasing the routine use of age appropriate, validated pain assessment measures (*e.g.*, Faces, Legs, Activity, Cry, and Consolability, FLACC [14]; Faces Pain Scale – Revised, FPS-R [15]; Numeric Rating Scale, NRS [16]; and Visual Analogue Scale, VAS [17]).

All units initially developed one pain practice change aim; 6/16 (38%) focused on improving pain management practices; 9/16 (56%) chose pain assessment, and 1/16 (6%) chose a combination of pain assessment and management. Seven units took on additional practice change(s) in subsequent intervention cycles, with a total of 23 discrete pain practice change aims developed by the final cycle. Two of the initial aims (on two different units) were abandoned (1 pain management aim and 1 combined pain assessment and management) and replaced with more focused aims, which better suited the pain practice needs of the units; 11/23 (48%) of the final aims focused on improving pain assessment and 12/23 (52%) on pain management practices (Table 2).

**Table 2:** **Intervention unit practice change aims (cycles 1–4)**

Unit	Aim 1	Baseline	% change aim	% achieved by cycle 4	Aim 2 (& 3)	Baseline	% change aim	% achieved by cycle 4	Comments
**1**	**Management**	**0%**	**75%**	**74%**	**Management**	**0%**	**80%**	**57%**	**Aim 2 added Cycle 3-4**
**2**	**Assessment**	**45%**	**80%**	**77%**	-		-	-	-
**3**	**Assessment**	**4%**	**50-80%**	**73%**	**Management**	**0%**	**25-35%**	**71%**	**Aim 2 added Cycle 3-4**
**4**	**Assessment**	**76%**	**50-90%**	**82%**	**Management**	**21%**	**25%**	**30%**	**Aim 2 added Cycle 3-4**
**5**	**Management**	**0%**	**50-80%**	**64%**	**Management**	**8%** ^*^	**80%**	**74%**	**Aim 2 added Cycle 4**
**6**	**Management**	**0%**	**50%**	**8%**	-		-	-	-
**7**	**Assessment**	**0%**	**80%**	**75%**	**Management**	**2%**	**80%**	**58%**	**Aim 2 added Cycle 2-4**
**8**	**Assessment**	**4%**	**100%**	**88%**	**Management**	**0%**	**75%**	**75%**	-
**9**	**Management**	**3%**	**50-40%**	**4%**	-		-	-	-
**10**	**Assessment**	**19%**	**50-35%**	**8%**	-		-	-	-
**11**	**Assessment**	**49%**	**75-90%**	**94%**	-		-	-	-
**12**	**Management**	**7%**	**100%**	**84%**	-		-	-	-
**13**	**Assessment**	**0%**	**50%**	**76%**	-		-	-	-
**14**	**Management**	**0%**	**35-80%**	**100%** ^**^	**Assessment 2 & 3**	**81% & 56%** ^**^	**80%**	**100% & 76%**	**Aim 1 abandoned and Aim 2 & 3 added Cycle 4**
**15**	**Assessment**	**21%**	**30-55%**	**66%**	-		-	-	-
**16**	**Assessment & Management**	**54%** ^*^	**30-40%**	**20%** ^**†**^	**Assessment**	**77%**	**40-85%**	**84%**	**Aim 1 abandoned Cycle 3**
									**Aim 2 added Cycle 2**

^*^Cycle 1.

^**^Cycle 3.

^†^Cycle 2.

**Figure 2:**
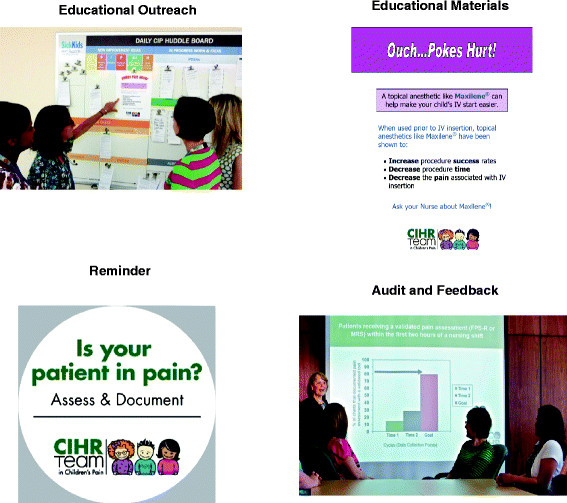
**Examples of knowledge translation strategies by type.**

In the Implementation and Change Phase of EPIQ, Research Practice Councils, in collaboration with the site research nurses, planned, developed, implemented and evaluated their practice change aims, employing evidence based KT strategies tailored to their unit, including: educational materials (*e.g.*, posters, newsletters), educational outreach activities (*e.g.*, one-on-one instruction, patient care rounds), reminders (*e.g.*, posters, stickers, buttons, screen savers), and audit and feedback (*e.g.*, surveys, chart audits, feedback reports). While other potential KT strategies exist, these four types were selected by the research team based on their effectiveness at the time of the study. All interventions used to achieve the practice change aims were evidence-based. Examples of KT strategies included: Educational posters based on Cochrane reviews of pain management interventions (*e.g.*, distraction, topical anesthetics), reminders about targeted practices strategically posted on the units or patient charts; laminated validated pain assessment scales placed at the patients’ bedside; educational instruction on the targeted pain practices in orientation sessions for new and returning staff; educational outreach to engage clinicians during reports on patient pain during patient care rounds; and summaries of high impact published articles on pain practices in unit newsletters (Figure 2).

Following each of the four intervention cycles, data were collected by the research nurses on the units’ progress in achieving their practice change aim from patient charts and using the Process Evaluation Checklist [11] for each KT strategy employed. The Research Practice Councils were provided with standardized feedback reports and discussed the usefulness of the KT strategies. This information informed the continuation, modification, or abandonment of pain practice change aims and KT strategies for the next intervention cycle. Some KT strategies were used throughout the intervention, such as laminated posters or checkbox reminders permanently added to patient documentation. Some strategies were updated to attract attention or replaced when worn out or lost. Strategies that were not considered useful or suitable to the unit were abandoned. The aims and KT strategies implemented were not only tailored and adapted to the identified needs of the units at the start of the study but were also reformulated based on the barriers and facilitators identified in each unit throughout the cycles of change.

### Study procedures and measures

Site investigators from the CIHR Team in Children’s Pain and research nurses at each site monitored and implemented the study processes; they attended a two-day EPIQ training session at the core site prior to the intervention. Site investigators were responsible for supporting the research nurses in their activities and attending key meetings with the study units. Research nurses assisted the intervention units’ health care professionals in the implementation of EPIQ and conducted data collection on all units. Research Ethics Board approval was obtained at the core site, The Hospital for Sick Children, and at each of the participating sites.

Immediately following each of the four EPIQ intervention cycles, data were collected on all of the intervention units. The research nurses and Research Practice Councils completed a Process Evaluation Checklist [11] for each KT strategy implemented during the intervention cycle. This checklist includes information on the KT strategies used, when and how often they were used, the number and type of individuals targeted for each strategy, the perceived usefulness of the strategies (rated on a 5-point Likert scale from ‘not at all useful’ to ‘extremely useful’), and any perceived barriers or facilitators to their implementation. Beginning support has been established for the content and construct validity, feasibility, and clinical utility of the Process Evaluation Checklist for use with complex interventions [11],[18]. Data were collected using this checklist from the Research Practice Council and, whenever possible, unit staff were consulted regarding the perceived usefulness of the KT strategies. The research nurses also reviewed approximately 30 patient charts per intervention unit following each cycle to determine the proportion of eligible patients (determined by the unit’s pain practice aim) who received the unit’s targeted pain practice(s) (*e.g.*, had their pain assessed using a validated age-appropriate measure), using the Rapid Cycle Evaluation Form, developed and pre-tested specifically for this study.

### Data analysis

Unit characteristics were described using means and standard deviations for continuous factors (*e.g.*, mean patient age on unit), and frequencies and percentages for categorical factors (*e.g.*, type of unit). The number of KT strategies implemented by type was summarized using medians given the nature of the data. The frequency and proportion of patients receiving targeted pain practices were summarized by intervention cycle. Logistic regression, using generalized estimating equations (*i.e.*, to account for clustering of patients within units), was used to model receipt of targeted pain practice onto cycle time period to determine if differences in the likelihood of receipt of pain practices existed over time. In separate analyses, the Kruskal-Wallis non-parametric test was used to analyze the influence of unit type on the median number of KT strategies used. Linear mixed models accounting for the correlation of multiple strategies within each unit were used to model the usefulness score of strategy on type of strategy.

To evaluate the effect that type and frequency of KT strategy had on attainment of targeted practice change aims, units were classified as to whether they came within 80% of their stated aim; this percentage was chosen as it was thought to be associated with clinical importance. Whether a unit achieved 80% or more of the stated aim was regressed on to the number of each type of KT strategy employed using logistic regression. Analyses were not adjusted for other factors given the small sample size available (*i.e.*, 16 units). Qualitative data collected on barriers and facilitators to the implementation of KT strategies from the Process Evaluation Checklist [11] were analyzed using content analysis methods [19]-[21]. Data were coded by the intervention cycle and according to KT strategy type and then categorized across type and cycle based on recurring themes.

## Results

### Effectiveness of evidence-based KT strategies

**Figure 3:**
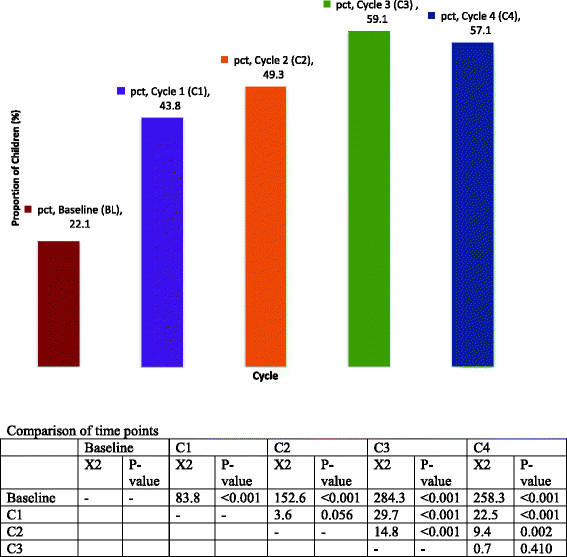
**Proportion of children receiving targeted pain practice per intervention cycle.**

At the completion of the four EPIQ intervention cycles: 18/23 (78%) pain practice aims were met, exceeded, or came within 80% of achievement, while 5/23 (22%) aims were less than 80%; 92% of the pain assessment aims were attained or came within 80% compared to 64% of the pain management aims. However this difference was not statistically significant (p = 0.16), in part due to the small sample size. Over the four EPIQ intervention cycles, a significant improvement in all selected pain assessment and pain management practices was observed. There was an absolute increase of 35% in the proportion of children receiving the targeted pain practices (22% at baseline versus 57% at the end of cycle four), regardless of the pre-determined practice aims or the degree of change units proposed (Figure 3); although a small and non-significant decrease (2%) in the number of patients receiving the targeted pain practices occurred between the 3rd (59.1%) and the final practice change cycle (57.1%).

### KT strategies implemented and their influence on pain practices

Over one-half of the units implemented 12 or more different reminders, six or more educational outreach sessions, seven or more educational materials, and seven or more audit and feedback strategies. The majority of strategies employed were reminders. The median number of strategies employed by units was 35, with an interquartile range from 21 to 47 strategies. Across the four EPIQ intervention cycles, there were no significant differences in how useful each type of KT strategy was rated using the Process Evaluation Checklist [11] (F_(3,504)_ = 0.63; p = 0.597); although educational outreach was rated the most useful strategy (Mean = 3.57 out of 5.0, SD = 2.12), followed by reminders (Mean = 3.50 out of 5.0, SD = 1.87), audit and feedback (Mean = 3.44 out of 5.0, SD = 2.67), and educational materials (Mean = 3.41 out of 5.0, SD = 2.95).

**Figure 4:**
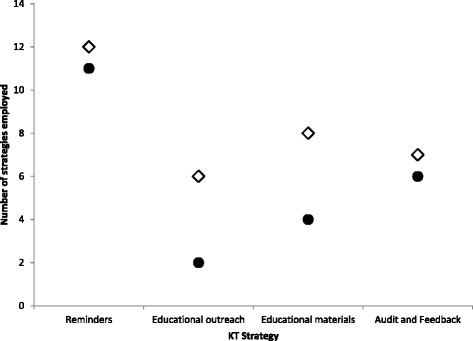
**Median cumulative number of knowledge translation strategies implemented by type†.** Note † comparing units (N = 16) that met aims by C4 vs those that did not**.** ◊ Units that met, exceeded, or came within 80% of their stated practice aim. ● Units that achieved less than 80% of their stated practice aims.

An overall increase in the number of strategies units employed increased the likelihood of achieving or coming within 80% of achieving their pain practice change aims (OR 1.13, 95% CI 1.02, 1.25; p = 0.022) (Table 3). Although the total number of strategies employed was higher for each strategy type among the units that achieved their aims (or within 80%) (Figure 4), the likelihood of achieving the aim did not statistically increase with greater numbers of strategies when examined by strategy type (Table 3). Furthermore, no statistically significant differences were found between type of unit (*e.g.*, medical, surgical, critical care) and the KT strategies implemented during the intervention implementation.

**Table 3:** **Logistic regressions modeling likelihood of achieving 80% or greater of unit practice aims by cycle 4 on number of strategies employed**

Factor	OR^a^(95% CI)	LR Chi-square (1 df)	P-value	c-statistic
Number of reminders used	1.06 (0.89 to 1.26)	0.49	0.513	0.54
Number of educational outreach strategies used	0.97 (0.79 to 1.20)	0.06	0.803	0.49
Number of educational materials used	1.14 (0.90 to 1.44)	1.61	0.272	0.68
Number of audit and feedback strategies used	1.10 (0.87 to 1.39)	0.75	0.438	0.51
Total number of strategies used	1.13 (1.02 to 1.25)	5.25	0.022	0.78

^a^Odds ratios are interpreted as the increase in odds of a unit achieving their aims for each additional strategy employed.

### Facilitators and barriers to the implementation of KT strategies

A number of factors were identified that facilitated the implementation of KT strategies across all strategy types and cycles of change:

1. staff engagement in the intervention, involvement in its implementation, and consensus on the practice change aim;
2. support and involvement of unit leadership (*e.g.*, dedicating staff time to implement strategies or participate in educational outreach, providing budget to produce reminders/educational materials);
3. assistance of the research nurse in the development, implementation, and maintenance of strategies;
4. resources to produce strategies and dedicated staff time to develop and implement them;
5. use of pre-existing systems of communication on the unit to deliver strategies and incorporating strategies into daily practice (*e.g.*, use of emails to reach all staff, posting reminders on flow sheets, reporting/facilitating discussion on targeted practice during rounds, scheduling presentations during educational days);
6. tailoring strategies to the unit in both form and content, with attention to unit culture and patient population; use of a variety of forms to deliver information (*e.g.*, verbal, posters, information sheets) and combining strategies (*e.g.*, using educational materials as tools in one on one instruction or presentations, reminders and audit and feedback providing opportunity for facilitated discussion).

### Characteristics of effective KT strategies

Specific visual, composition, and other delivery characteristics enhanced the effective implementation of KT strategies. Reminders and educational materials were reported to be more effective when they were colourful, eye catching and large; durable, well-located, and easily accessible (*e.g.*, at points of care, on/in patient charts, at nursing station); novel and creative; practical and easy to deliver; and included images and visual cues. Effective educational materials were clear and concise, easy to read and understand, informative, relevant and useful for practice. Audit and feedback strategies were facilitated by including graphs and diagrams, following up with discussion of practice change, and demonstrating progress/change over time.

Scheduling was an important factor in educational outreach, ensuring the availability of staff to participate, the inclusion of experts in presentations, and unit leader presence to enhance credibility.

Several barriers in the implementation of strategies were also identified. These included:

1. competing priorities on the unit, such as workload, patient acuity, staff shortages, or other initiatives at the unit or hospital levels, as well a major disease outbreak during the intervention period;
2. lack of staff availability (*e.g.*, to participate in educational outreach or read educational materials);
3. inadequate time for the development and delivery of strategies (*e.g.*, stickers posted daily on patient charts/flow sheets; laminated pain scales attached to bedside; poster on sucrose in rooms of eligible patients);
4. inconsistent delivery or use of strategies (*e.g.*, reminders and educational materials were displaced or removed and not replaced; stickers not added to all patient charts);
5. limited reach to all staff (*e.g.*, staff did not read emails, attend meetings);
6. lack of clarity or sufficient information in educational materials and reminders; too wordy, too small to be useful; format not well received/appropriate for unit (*e.g.*, staff didn’t like to wear buttons, image on poster disturbed parents); not well located;
7. decrease in staff interest and engagement over time (*e.g.*, ‘staff were getting tired of hearing about sucrose’; posters ‘blended in’ and were no longer noticed or read over time, strategies lost impact over time and novelty ‘wore off’).

## Discussion

### Effectiveness of evidence-based KT strategies

In this national, multi-site study, the EPIQ intervention resulted in significant achievement of pain practice change aims (78% of targeted aims came within 80% of achievement) and overall improved pain assessment and management on the participating units. Regardless of the selected pain practice aims on EPIQ units, overall, children were increasingly more likely to receive the targeted pain practices across the intervention cycles. There was, however, a small decrease in the overall use of targeted pain practices in the final cycle. This decrease may indicate a plateau effect or intervention fatigue. Competing priorities or events, which occurred in the practice context, could also have contributed to this reduction in pain practices, such as the introduction of a new practice guideline at the institutional level, resulting in the need to change procedures or processes on the unit [18].

The positive impact of multifaceted, evidence-based KT strategies, implemented to achieve pain practice change, contributes to growing evidence supporting the effectiveness of multifaceted KT interventions [18],[22]. An increase in the total number of KT strategies implemented increased the likelihood of units achieving their pain practice change aims. This finding may be explained by both an interdependence and cumulative effect of the combination of KT strategies implemented over the course of the study. However, Grimshaw and colleagues [1],[3] reported that in multifaceted interventions, effect sizes did not necessarily increase with the number of KT strategies implemented.

### KT strategies implemented

While all intervention units followed the same process of iterative intervention cycles in the implementation of change, the KT strategies employed to achieve pain practice aims varied from unit to unit and cycle to cycle. Each unit planned, developed, and implemented strategies tailored to their unit’s culture and needs, and retained, revised, and/or abandoned them based on the evaluation of their effectiveness and feedback from staff. Reminders emerged as the most commonly employed strategy; however, no significant differences were found in the perception of their usefulness to influence change across the type of KT strategies implemented (based on results from the Process Evaluation Checklist [11]), nor in their effect on pain assessment and management practices. Furthermore, an optimal ‘dose’ and/or combination of specific KT strategies required to achieve pain practice change was not identified. Yamada *et al.*[18] were similarly unable to identify an ‘optimal dose’ or combination of strategies required for pain practice change using EPIQ in the neonatal intensive care unit.

The four types of KT strategies employed in this study do not encompass the full range of potential KT interventions and others may need to be considered. For example, the use of local opinion leaders has been shown to promote improvements in care [3],[23]. These informal leaders can influence the behaviours of others within a social network [2],[23]. Many of the Research Practice Council members in this study could be defined as local opinion leaders in the participating units; however, some members were chosen by their unit managers and were not self-selected champions. The role of Research Practice Council members in pain practice change and the degree of their influence on process and clinical outcomes requires future investigation.

### Barriers and facilitators to implementation of KT strategies

Units that were successful in achieving their aims employed more KT strategies than units that did not. However, contextual factors, unique to each unit and site, may have moderated the ability of a unit to achieve pain practice change. Factors such as staff time and engagement, leadership support, and systems of communication may have influenced a unit’s success. Squires *et al.*[24] similarly found that contextual factors such as these may be influential in successful translation of evidence into practice. The impact of context on the effectiveness of multifaceted KT interventions requires further consideration in future research.

The quality of the KT strategies implemented and consistency in their implementation may have influenced their impact on change. Stevens *et al.*[8] suggest that the strength of the EPIQ intervention in achieving pain practice change, as well as improved clinical outcomes, is in its tailored design, with aims customized to the unit’s most pressing pain problems and strategies selected based on unit preferences and readiness for change. This tailoring aimed to better address barriers to practice change [25]. In a systematic review of the effectiveness of tailored interventions, Baker and colleagues [6] reported that interventions that addressed barriers to change were more likely to improve practice outcomes compared to no intervention or a more passive dissemination of guidelines. Throughout the study, evidence-based pain practice change aims and KT strategies were developed and implemented by Research Practice Council members at the unit level, informed by local evidence, and addressing the unique needs and culture of the unit. These considerations are consistent with the Promoting Action on Research Implementation in Health Services (PARiHS) framework [26],[27]. According to this framework, the implementation of evidence into practice is successful when strong research and local evidence are used to determine and guide change, influential and skilled facilitators support change, and there is a positive practice context (*e.g.*, leadership support) [26],[27].

### Limitations and sustainability

The planning, development, and implementation of multifaceted KT strategies require support from unit leadership, dedicated staff time, and financial resources. In this study, the research nurses assisted pain practice, health care professional champions throughout the process and each unit received a small amount of funding for the development of unique KT strategies. The sustainability of the improved pain practices and the continued use of KT strategies to achieve practice change without the support of the research nurse or study resources are unknown. In a recent casebook on the experiences of the research nurses of the implementation of EPIQ, a number of the strategies used were identified as costly and/or labour intensive to create and maintain [28].

## Conclusions

The overall aim of this study was to improve pain assessment and management over time. Multifaceted, tailored KT interventions may be the first step in promoting optimal pain practices; however whether or not they are the answer to sustained improvement is unknown. Research is required to evaluate the effectiveness of KT strategies over time and sustainability of these pain practice changes when more formal research supports, such as research nurses are not available. Additional factors, such as the complex organizational structures in which the majority of clinicians work, institutional policies, and leadership support, can exert significant influence on the success of planned practice changes and require additional evaluation. Further research is also required to evaluate the use of KT strategies in less resource intensive sites, such as community or rural hospital settings, and factors related to the quality and consistency of the implementation process, such as feasibility and fidelity.

## Authors’ original submitted files for images

Below are the links to the authors’ original submitted files for images. Authors’ original file for figure 1 Authors’ original file for figure 2 Authors’ original file for figure 3 Authors’ original file for figure 4

## Abbreviations

KT: Knowledge translation
EPIQ: Evidence-based Practice for Improving Quality

## References

[1] Grimshaw JM, Thomas RE, MacLennan G, Fraser C, Ramsay CR, Vale L, Whitty P, Eccles MP, Matowe L, Shirran L, Wensing M, Dijkstra R, Donaldson C (2004). Effectiveness and efficiency of guideline dissemination and implementation strategies. Health Technol Assess.

[2] Scott-Findlay SD, Estabrooks C, Finley GA, McGrath PJ, Chambers CT (2006). Knowledge translation and pain management. Bringing Pain Relief to Children.

[3] Grimshaw JM, Eccles MP, Lavis JN, Hill SJ, Squires JE (2012). Knowledge translation of research findings. Implement Sci.

[4] Prior M, Guerin M, Grimmer-Somers K (2008). The effectiveness of clinical guideline implementation strategies-a synthesis of systematic review findings. J Eval Clin Pract.

[5] Hakkennes S, Dodd K (2008). Guideline implementation in allied health professions: a systematic review of the literature. Qual Saf Health Care.

[6] Baker R, Camosso-Stefinovic J, Gillies C, Shaw EJ, Cheater F, Flottorp S, Robertson N (2010). Tailored interventions to overcome identified barriers to change: effects on professional practice and health care outcomes. Cochrane Database Syst Rev.

[7] Lee SK, Aziz K, Singhal N, Cronin CM, James A, Lee DS, Matthew D, Ohlsson A, Sankaran K, Seshia M, Synnes A, Walker R, Whyte R, Langley J, MacNab YC, Stevens B, von Dadelszen P (2009). Improving the quality of care for infants: a cluster randomized controlled trial. CMAJ.

[8] Stevens BJ, Yamada J, Estabrooks CA, Stinson J, Campbell F, Scott SD, Cummings G (2013). Pain in hospitalized children: effect of a multidimensional knowledge translation strategy on pain process and clinical outcomes. Pain.

[9] Stevens BJ, Abbott LK, Yamada J, Harrison D, Stinson J, Taddio A, Barwick M, Latimer M, Scott SD, Rashotte J, Campbell F, Finley GA (2011). Epidemiology and management of painful procedures in children in Canadian hospitals. CMAJ.

[10] Stevens BJ, Harrison D, Rashotte J, Yamada J, Abbott LK, Coburn G, Stinson J, Le May S (2012). Pain assessment and pain intensity of children in Canadian pediatric hospitals. J Pain.

[11] Yamada J, Stevens B, Sidani S, Watt-Watson J (2010). Content validity of a process evaluation checklist to measure intervention implementation fidelity. Worldviews Evid Based Nurs.

[12] Langely GJ, Nolan KM, Norman CL, Provost L (1996). The improvement guide: a practical approach to enhancing organizational performance.

[13] Taylor MJ, McNichols C, Nicolay C, Darzi A, Bell D, Reed JE (2014). Systematic review of the application of the plan-do-study-act method to improve quality in healthcare. BMJ Qual Saf.

[14] Merkel SI, Voepel-Lewis T, Shayevitz JR, Malviya S (1997). The FLACC: a behavioral scale for scoring postoperative pain in young children. Pediatr Nurs.

[15] Hicks C, von Baeyer C, Spafford P, van Korlaar I, Goodenough B (2001). The faces pain scale-revised: toward a common metric in pediatric pain measurement. Pain.

[16] Jensen MP, Karoly P, Braver S (1986). The measurement of clinical pain intensity: a comparison of six methods. Pain.

[17] Scott P, Ansell B, Huskisson E (1977). Measurement of pain in juvenile chronic polyarthritis. Ann Rheum Dis.

[18] Yamada J, Stevens B, Sidani S, Watt-Watson J, Sidani S: Test of a process evaluation checklist to improve neonatal pain practices.*West J Nurs Res* 2014, [Epub ahead of print]., 10.1177/0193945914524493 24577869

[19] Cole FL (1988). Content analysis: process and application. Clin Nurs Spec.

[20] Elo S, Kyangäs H (2008). The qualitative content analysis process. J Adv Nurs.

[21] Hsieh H, Shannon S (2005). Three approaches to qualitative content analysis. Qual Health Res.

[22] Zhu LM, Stinson J, Palozzi L, Weingarten K, Hogan ME, Duong S, Carbajal R, Campbell FA, Taddio A (2012). Improvements in pain outcomes in a Canadian pediatric teaching hospital following implementation of a multifaceted knowledge translation initiative. Pain Res Manag.

[23] Flodgren G, Parmelli E, Doumit G, Gattellari M, O’Brien MA, Grimshaw J, Eccles MP (2011). Local opinion leaders: effects on professional practice and health care outcomes. Cochrane Database Syst Rev.

[24] Squires JE, Estabrooks CA, Scott S, Cummings G, Hayduk L, Kang SH, Stevens B (2013). The influence of organizational context on the use of research by nurses in Canadian pediatric hospitals. BMC Health Serv Res.

[25] Wensing M, Bosch M, Grol R, Straus S, Tetroe J, Graham ID (2013). Developing and selecting knowledge translation interventions. Knowledge translation in health care.

[26] Rycroft-Malone J, Rycroft-Malone J, Bucknall T (2010). Promoting Action on Research Implementation in Health Services (PARiHS). Models and Frameworks for Implementing Evidence-Based Practice: Linking Evidence to Action.

[27] Kitson A, Harvey G, McCormack B (1998). Enabling the implementation of evidence based practice: a conceptual framework. Qual Health Care.

[28] Widger K, Barwick M, Stevens B (2013). Stories from the floor: a knowledge translation casebook on improving pediatric pain practices. ᅟ.

