# Supplementary figures and images for: Implementation of multidimensional knowledge translation strategies to improve procedural pain in hospitalized children
Source: Implement Sci. 2014 Nov 25;9:120. doi: 10.1186/s13012-014-0120-1 (PMC4263210; doi:10.1186/s13012-014-0120-1)

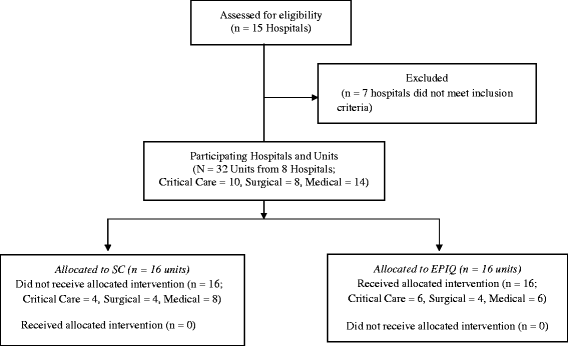

Supplement: Supplementary file 1 — Authors’ original file for figure 1 [file 13012_2014_120_MOESM1_ESM.gif]

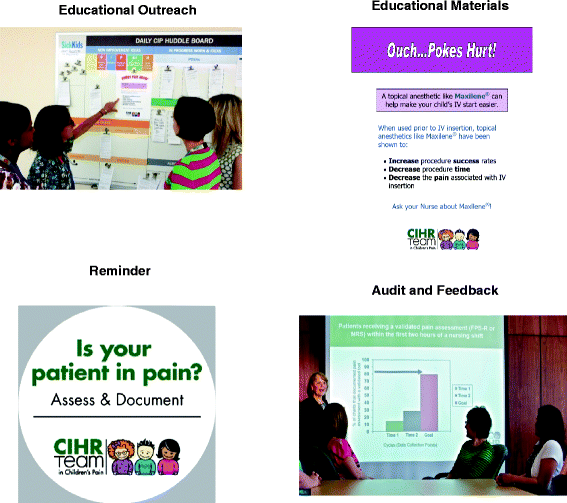

Supplement: Supplementary file 2 — Authors’ original file for figure 2 [file 13012_2014_120_MOESM2_ESM.gif]

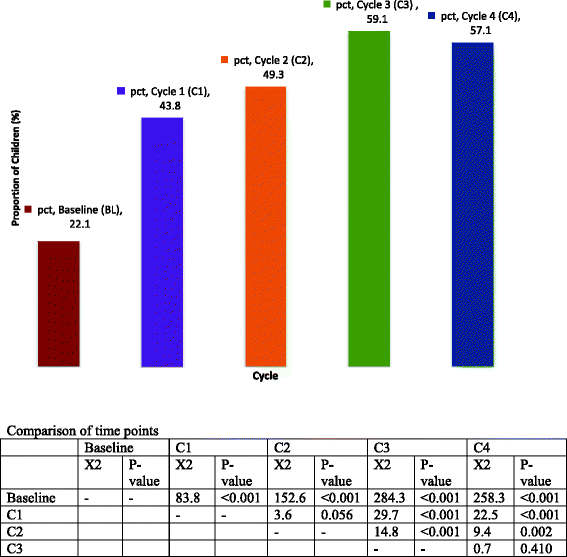

Supplement: Supplementary file 3 — Authors’ original file for figure 3 [file 13012_2014_120_MOESM3_ESM.gif]

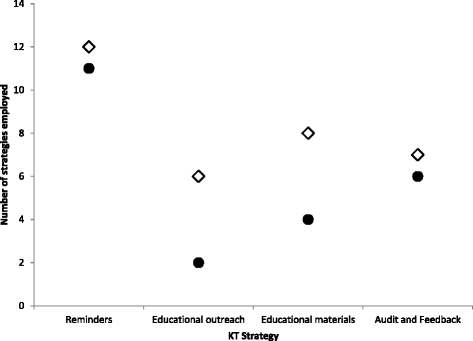

Supplement: Supplementary file 4 — Authors’ original file for figure 4 [file 13012_2014_120_MOESM4_ESM.gif]
